# Evaluation of acetylation and methylation in oral rinse of patients with head and neck cancer history exposed to valproic acid

**DOI:** 10.1038/s41598-021-95845-3

**Published:** 2021-08-12

**Authors:** Ricardo Ribeiro Gama, Lidia Maria Rebolho Batista Arantes, Bruna Pereira Sorroche, Pedro De Marchi, Matias Eliseo Melendez, Raiany Santos Carvalho, Marcos Alves de Lima, André Luiz Vettore, André Lopes Carvalho

**Affiliations:** 1 Head and Neck Surgery Department, Barretos Cancer Hospital, Rua Antenor Duarte Villela, 1331, Bairro Dr. Paulo Prata, Barretos, SP 14784-400 Brazil; 2 Postgraduation Program, São Paulo Federal University, São Paulo, SP Brazil; 3 Molecular Oncology Research Center, Barretos Cancer Hospital, Barretos, SP Brazil; 4 Clinical Oncology Department, Barretos Cancer Hospital, Barretos, SP Brazil; 5 Oncoclínicas, Rio de Janeiro, RJ Brazil; 6 Researcher Support Center, Teaching and Research Institute, Barretos Cancer Hospital, Barretos, SP Brazil; 7 Biostatistics Center, Teaching and Research Institute, Barretos Cancer Hospital, Barretos, SP Brazil; 8 Cancer Molecular Biology Laboratory, São Paulo Federal University, Diadema, SP Brazil; 9 Senior Researcher, International Agency for Research on Cancer (IARC), Lyon, France

**Keywords:** Cancer, Chemical biology, Molecular biology, Oncology

## Abstract

Evaluate the biological action of valproic acid in the acetylation of histones and in the methylation of tumor suppressor genes via oral rinse in patients with a previous history of head and neck squamous cell carcinoma (HNSCC). Forty-two active or former smokers were included in this randomized, double-blind, placebo-controlled trial. Oral rinse samples were collected prior to treatment with valproic acid or placebo and after 90 days of treatment. The methylation status of five tumor suppressor genes and histone acetylation were evaluated by pyrosequencing and ELISA techniques, respectively. Differences between the 90-day and baseline oral rinse acetylation and methylation results were analyzed by comparing groups. Thirty-four patients were considered for analysis. The mean percentage adherence in the valproic and placebo groups was 93.4 and 93.0, respectively (p = 0.718). There was no statistically significant difference between groups when comparing the medians of the histone acetylation ratio and the methylation ratio for most of the studied genes. A significant reduction in the *DCC* methylation pattern was observed in the valproic group (p = 0.023). The use of valproic acid was safe and accompanied by good therapeutic adherence. *DCC* methylation was lower in the valproic acid group than in the placebo group.

## Introduction

Head and neck cancer (HNC) is the seventh most common cancer and the fifth most frequent among men, accounting for 888,000 new cases worldwide in 2018^1^, and is associated with 453,000 deaths. Males are affected three times as often as females, and 70% of new cases and 75% of deaths occur in middle- and low-income countries^2^.

Epigenetic events such as cytosine methylation in cytosine-guanine (CpG) dinucleotides and modification of histones such as acetylation and deacetylation are considered early carcinogenic events in head and neck squamous cell carcinoma (HNSCC) as a result of smoking and alcoholism^3–6^. DNA methylation and histone modification by acetylation or deacetylation act together in chromatin remodeling, the inactivation of suppressor genes and the activation of oncogenes^5,7^. DNA methyltransferases (DNMT) and histone deacetylases (HDAC) are enzymes that act as mediators of epigenetic events, methylating DNA and deacetylating histones, respectively. HDAC inhibitors represent a new class of tumor inhibitors capable of affecting multiple genes and molecular pathways based on the function of the epigenetic enzymes they regulate through the acetylation of histone and nonhistone proteins. By altering the chromatin structure, HDAC inhibitors influence gene transcription, DNA repair and replication mechanisms^8^.

Valproic acid, commonly used in clinical practice as an anticonvulsant and mood stabilizer, is reported to be a class 1 and 2 HDAC inhibitor promoting hyperacetylation of the N-terminal chains of histones H3 and H4 and nonhistone proteins. In addition, some authors report that an increase in HDAC function leads to the stabilization of DNMT and that valproic acid can degrade DNMT1 through various biochemical mechanisms, including acetylation^9^.

By promoting the acetylation of histones H3 and H4 through HDAC inhibition, valproic acid modifies the chromatin structure, avoiding the inhibition of tumor suppressor gene expression. In a preclinical study, Gan et al.^10^ found that valproic acid inhibited the growth of HNSCC cell lines at physiological doses.

Because of its HDAC inhibitory activity and because it has been recognized for years as a safe treatment for epilepsy, valproic acid has been considered a good candidate for anticancer treatment in patients with metastatic or recurrent HNC.

Certain clinical trials for HNSCC have included the use of valproic acid in association with conventional treatment to increase the rate of therapeutic response^11,12^. Kang et al.^4^ conducted a prospective study evaluating the risk of developing several malignant tumors in relation to the use of valproic acid through the collection of clinical and epidemiological information in the computerized database of the National Veterans Affairs (VA) together with data from the VA Central Cancer Registry. This study showed that patients over 40 years old with a history of smoking and who used valproic acid for at least 1 year due to neurological or psychiatric diseases showed a 34% reduction in the risk of developing head and neck cancer. They also concluded, in a multivariate analysis, that this significant reduction in the development of HNC in valproic acid users in a high-risk group was associated with the length of administration of the drug (when greater than three years, there was a significant reduction in the risk of HNC) and its serum levels (reached with the therapeutic dose necessary to obtain a serum drug value greater than or equal to 40 µg/mL).

In view of the above, the aim of this study was to verify the biological action of valproic acid in the acetylation of histones and in the methylation of tumor suppressor genes in oral rinse samples of patients with a previous history of HNSCC.

## Methods

### Type of study

Randomized, prospective, double-blind, placebo-controlled clinical trial. This clinical trial took place at Barretos Cancer Hospital in a joint cosponsorship from the Head and Neck Surgery Department and the Clinical Research Unit from January to December 2016. Molecular analyses of the material derived from the clinical trial were performed at the Molecular Oncology Research Center.

### Ethical considerations

This study was evaluated by the Research Ethics Committees of the Barretos Cancer Hospital (BCH) and the Federal University of São Paulo and was approved under numbers 1,805,064 and 3,378,445, respectively. The research was performed in accordance with the relevant guidelines and regulations. Informed consent was obtained from all participants before the inclusion and randomization processes. All the biological samples analyzed were collected (four per patient) and stored in an institutional biorepository at the BCH. Every procedure for identifying the samples followed a careful process so that there was no breach of confidentiality of personal information. This research was registered on the Clinical Trials website under number NCT02608736 in 20/11/2015, URL: https://clinicaltrials.gov/ct2/show/NCT02608736

This clinical trial was also registered at the WHO International Clinical Trial Platform with registration number 11039 on 27/01/2021.

### Study population

The selected patients were followed-up at the outpatient clinic of the Head and Neck Surgery Department, and their recruitment occurred at random, as long as there was no clinical and/or radiological evidence of neoplasia. A total of 42 patients were randomized.

### Inclusion and exclusion criteria

The main inclusion criteria were age over 18 years and a previous history of squamous cell carcinoma of the oral cavity, oropharynx, larynx or hypopharynx treated at the institution with curative intent and in active outpatient follow-up, having completed the treatment between 3 and 36 months and without evidence of active disease at the time of inclusion. Regarding the ECOG (Eastern Cooperative Oncology Group) clinical performance scale, the patient should be classified with a score between 0 and 2 and have adequate hematological, renal and hepatic function, in addition to having all toxicities attributed to previous anticancer therapy resolved for Grade 1—CTCEA NCI version 5^13^ or for the baseline.

The exclusion criteria were as follows: nonsmoking patients; patients considered dependent on alcohol consumption; those already using valproic acid for another pathology; and those with selected comorbidities such as HIV, hepatitis B, epilepsy, a psychiatric disorder that required drug treatment or those already using drugs that could cause an interaction in the central nervous system.

### Experimental design

#### Study groups and doses of the drug used

The randomization process was stratified by smoking status, which balanced the distribution of active and former smokers between the arms of the study.

Patients received placebo or magnesium valproate (valproic acid) for three months via capsules prepared by the company *Manipulart,* located in Barretos, Brazil. Patients weighing less than 55 kg used 1000 mg/day valproic acid divided into two 500 mg doses every 12 h. Patients weighing 55 kg or more used 1500 mg/day valproic acid divided into two doses, 500 mg in the morning and 1000 mg at night, at a 12 h interval. In the first week, they received 500 mg/day, and after 7 days, they received a weekly increase of 500 mg/day until they reached the programmed dose. Those under 55 kg received a weekly increase of 250 mg/day after the first week.

#### Biological validation

For validation of the biological action of valproic acid in oral rinse samples, acetylation of histones H3 and H4 and methylation of Cyclin A1 (*CCNA1*), Cyclin Dependent Kinase Inhibitor 2A or P16INK4A (*CDKN2A*), Death Associated Protein Kinase (*DAPK*), Deleted Colorectal Carcinoma (*DCC*), *O*-6-Methylguanine-DNA Methyltransferase (*MGMT*) and Long Interspersed Long Element 1 (LINE-1—repetitive sequence which infers global methylation) were evaluated by ELISA and pyrosequencing, respectively.

All genes evaluated in this research are tumor suppressors, and their silencing could contribute to the process of carcinogenesis^14^. Among the studied genes are *CCNA1*^15^, *DAPK*^16^ and *DCC*^17^, which are involved in the cell cycle and apoptosis; *MGMT*^16,18^ is associated with DNA repair; and *CDKN2A*^16,18^ acts in the control of the cell cycle. It has been verified that the expression of these genes can be affected by aberrant methylation of their promoter region in association with transcriptional silencing in HNSCC^14^.

These genes had been previously studied by our group and were reported to be frequently methylated in tissue, salivary rinse and exfoliated cells collected from HNSCC patients and rarely methylated in samples collected from noncancer individuals^14,17,19–21^. The finding that these genes were specifically methylated in biological samples from HNSCC patients suggests their role in the carcinogenesis of these tumors.

When analyzing acetylation status between the valproic and placebo groups, the ratio calculated by dividing the acetylation value of histones H3 and H4 in posttreatment oral rinse samples over the acetylation of histones H3 and H4 in pretreatment oral rinse samples was considered. Regarding the analysis of the methylation profile of the valproic and placebo groups, the ratio was calculated by the methylation value for each of the genes in posttreatment oral rinse samples divided by the methylation of these same genes in pretreatment oral rinse samples.

##### Sample collection and DNA extraction

Oral rinses were obtained by mouth rinsing with 5 mL of normal saline solution (NaCl 0.9%) at two time points: on the day of randomization and after 90 days of treatment with placebo or valproic acid. Samples were centrifuged for 10 min at 1500 rpm at 4 °C, and cell pellets were stored at − 80 °C. DNA extraction from exfoliated cells present in oral rinses was carried out using a QIAamp DNA Micro Kit (*Qiagen*) according to the manufacturer’s instructions.

##### Bisulfite treatment

DNA from salivary rinses was subjected to bisulfite treatment as described previously^14^. Sodium-bisulfite conversion of 500 ng of DNA was performed using an EpiTect Bisulfite Kit (*Qiagen*) following the manufacturer’s recommendations.

##### Quantitative pyrosequencing methylation assay: PMA

Pyrosequencing was performed using a PyroMark Q24 sequencer (*Qiagen*) with a PyroMark Gold Q96 Reagent Kit (*Qiagen*) according to the manufacturer's protocol. A mean methylation index was calculated from the CpG site methylation percentages for each gene. The results were analyzed using default software settings. We considered 0–5% unmethylated (hypomethylated), and values above 5% were considered methylated (hypermethylated)^22^. The pyrosequencing primers are available upon request.

##### H3 and H4 histone acetylation analysis

H3 and H4 histone acetylation was evaluated by enzyme-linked immunosorbent assay (ELISA) using a Histone H3 Acetylation Assay Kit and Histone H4 Acetylation Assay Kit (*Abcam*), respectively, following manufacturer recommendations. Protein concentration was measured using a Bradford assay (*Thermo Scientific*). Samples were stored at − 80 °C. Fifteen micrograms of histone were assayed for each pre and posttreatment sample. Histone posttreatment/pretreatment ratios for both arms of the clinical trial are expressed as percentages.

### Statistical analysis

Descriptive data analysis was performed by calculating the mean and standard deviation or the median and minimum/maximum values for quantitative variables and with tables of absolute and relative frequency for qualitative variables.

To verify homogeneity between groups, sociodemographic and clinical variables were compared using the chi-square test or Fisher's exact test for qualitative variables and Student's t test for quantitative variables. The comparison of intragroup therapeutic adherence was performed using the Bonferroni test, and the incidence of adverse events was compared between groups using the difference in proportions test. The analysis of the medians of the methylation ratios of different genes in the posttreatment oral rinse (T1) in relation to the pretreatment time (T0) between the valproic and placebo groups was performed using the Mann–Whitney U test. The same test was used to compare the medians of the H3 and H4 histone acetylation ratios in the oral rinses between the groups. Significance was considered for a p-value < 0.05, and SPSS v21 Software was used for all analyses.

## Results

### Description of the sociodemographic and clinical characteristics of the study population

Participants were selected by means of stratified randomization by active tobacco consumption in the valproic or placebo groups and were approached on the day they attended the medical consultation without any prior invitation to participate in the research. After carefully checking the clinical inclusion criteria, 81 participants were approached and invited to participate in the study. Of these, 53 terms of consent were signed, while the remaining 28 refused to participate. Further assessment showed that 6 participants did not meet the necessary laboratory inclusion criteria, and with the withdrawal of 5 participants, 42 patients were double-blind randomized: 21 in the valproic group and 21 in the placebo group. Because of the suspension of treatment due to an adverse event or withdrawal from continuing the study, which led to the absence of biological material in the second period of collection previously established, eight patients had to be removed from the clinical trial; therefore, 34 participants were considered for the molecular analyses, 17 from the valproic group and 17 from the placebo group (Table 1). Specifically, regarding methylation analysis, we obtained 13 samples from the placebo group and 15 samples from the valproic acid group because there were not enough biological samples to study methylation from 4 and 2 participants in the placebo and valproic acid groups, respectively.

**Table 1:** Sociodemographic and clinical characteristics of the study population (n = 34).

Variables	Valproic acid group (n = 17) N (%)	Placebo group (n = 17) N (%)	*p* value^#^
**Sex**			1.000
Male	16 (94.1)	17 (100)	
Female	1 (5.9)	0 (0)	
**Ethnicity**			0.492
Caucasian	8 (47)	10 (58.9)	
Non-Caucasian	9 (53)	7 (41.1)	
**Previously treated primary tumor location**			0.283
Oral cavity	7 (41.1)	2 (11.8)	
Oropharynx	3 (17.7)	5 (29.4)	
Larynx	5 (29.4)	6 (35.3)	
Hypopharynx	2 (11.8)	4 (23.5)	
**ECOG**			1.000
1	13 (76.4)	14 (82.3)	
2	4 (23.6)	3 (17.7)	
**Tobacco status**			0.714
Active smoker	6 (35.3)	5 (29.4)	
Former smoker	11 (64.7)	12 (70.6)	
**Comorbidities**			1.000
Absent	15 (88.2)	16 (94.1)	
Present	2 (11.8)	1 (5.9)	

*ECOG* Eastern Cooperative Oncologic Groups.

^#^Calculated by Fisher's exact test except for tobacco status and ethnicity, which were calculated by the chi-square test.

Table 1 shows the sociodemographic characteristics of the study population. The patients were mainly males (97%), with ages ranging from 42 to 82 years (median 62 years), and 52.9% were Caucasian. Current and former tobacco consumption were self-reported by 32.3% and 67.7%, respectively. The previously treated primary tumor sites were in the oral cavity (67.7%), oropharynx (26.5%), larynx (32.3%) and hypopharynx (17.6%). An ECOG value of 1 was present in 79.4% of the patients, and comorbidities were present in 8.8% of the patients.

Additionally, the mean age in the valproic group was 57.0 (± 7) years, and that in the placebo group was 57.0 (± 10) years (p = 0.263). The mean packs/year consumed in the valproic and placebo group was 60.5 (± 48.9) and 40.7 (± 33.9), respectively (p = 0.201).

### Analysis of treatment interruptions, adherence and adverse events

Treatment interruptions in the study were computed and classified as temporary or definitive. Four participants (23.5%) in the valproic group had their treatment interrupted, 2 temporarily (11.7%) due to an adverse event related to the drug and to an unrelated adverse event, and the other 2 definitively (11.7%) due to a serious adverse event related to the drug and to tumor recurrence. There were no treatment interruptions in the placebo group; nevertheless, there was no difference between the groups (p = 0.103). There was poor adherence to treatment by one participant in the valproic group (p = 1.000).

Therapeutic adherence was assessed indirectly by counting the medications at intervals of 28–30 days at each of the three outpatient visits. The mean percentage adherence in the valproic and placebo group was 93.4 (± 7.4) and 93.0 (± 9.3), respectively (p = 0.718). In the analysis of intragroup adherence in the valproic arm, a mean percentage adherence of 88.5 (± 2.4) was observed 30 days after the use of the drug, 93.7 (± 2.6) at 60 days and 97.9 (± 2.7) at 90 days; the adherence at 90 days was significantly greater than that at 30 days (p = 0.031). There was no difference in intragroup adherence at different times in the placebo group (p = 0.631). Table 2 illustrates how adherence to treatment increased in the valproic group over time (time 30 (30 days of treatment), time 60 (60 days of treatment) and time 90 (90 days of treatment)) and how this adherence differed between times 30 and 90.

**Table 2:** Comparison of adherence (in p values) between time points in the valproic group (in days after randomization).

		Time 30	Time 60	Time 90
Valproic group	Time 30 *88.5 (± 2.4) n = 17	–	0.446	**0.031**
	Time 60 *93.7 (± 2.6) **n = 15	0.446	–	0.790
	Time 90 *97.9 (± 2.7) ***n = 14	**0.031**	0.790	–

*Mean percentage (standard deviation percentage).

**2 unaccounted participants: one for not bringing the vial for accounting and another for having the treatment definitively interrupted 47 days after randomization due to tumor recurrence.

***3 unaccounted participants: one for having the treatment definitively interrupted at the previous time point due to tumor recurrence, another for having poor adherence in the third month of treatment and the third for having the treatment definitively interrupted 56 days after randomization due to a serious drug-related adverse event.

Tabulated results represent p values calculated by the Bonferroni test. p-values < 0.05 are presented in bold.

Among the most common adverse events reported during the treatment period, we highlighted somnolence, increased AST/ALT levels, dizziness, asthenia, vomiting, anorexia, and dyspepsia (Table 3). Dyspepsia was more prevalent in the valproic group (p = 0.004). When analyzing the report of dyspepsia by time in the valproic group, 8 participants (47%), 6 participants (35.2%) and 4 participants (23.5%) reported it at 30, 60 and 90 days of treatment, respectively. Nevertheless, there was no significant difference in reporting this event when comparing times in the valproic group (p = 0.5 between 30 and 60 days, p = 0.219 between 30 and 90 days and p = 0.625 between 60 and 90 days).

**Table 3:** Main adverse events of the study in decreasing order of reporting.

Adverse event	Valproic acid group N (%)	Placebo group N (%)	p*
Somnolence	8 (47.0)	4 (23.5)	0.151
Dyspepsia	9 (52.9)	0 (0.0)	**0.004**
Increased AST/ALT levels	1 (5.8)	2 (11.7)	1.000
Dizziness	1 (5.8)	2 (11.7)	0.310
Asthenia	2 (11.7)	0 (0.0)	0.145
Vomiting	1 (5.8)	1 (5.8)	0.545
Anorexia	1 (5.8)	0 (0.0)	0.545

*Calculated by the proportion difference test. p-value < 0.05 is presented in bold.

### Acetylation analyses

**Figure 1:**
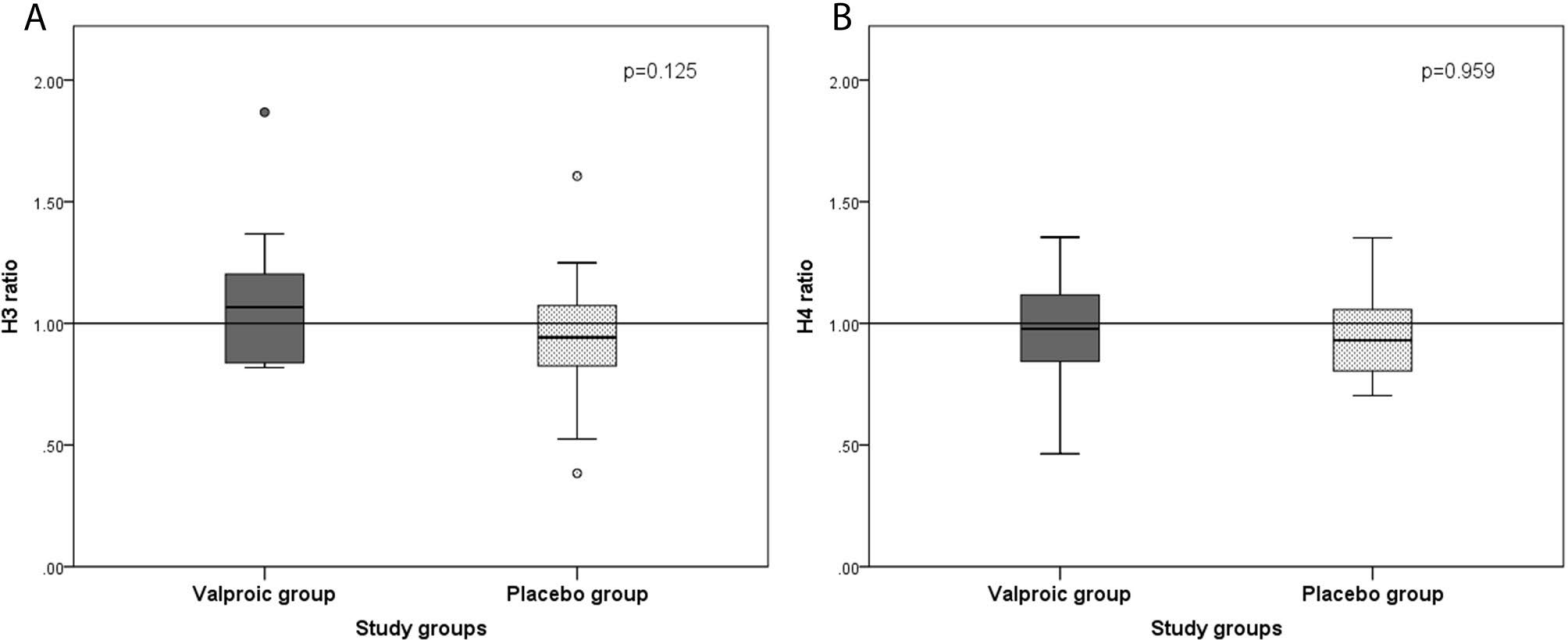
Comparison of the medians of the T1/T0 histone acetylation ratios H3 (**A**) and H4 (**B**) in oral rinse samples between the valproic and placebo groups. The boxplots presented above show the median, maximum and minimum values, interquartile range and outliers (represented by circles) in the two study groups. The dashed line passing through 1 indicates that the group participants located above the line were acetylated.

Regarding the acetylation of histones H3 and H4 in the oral rinse, it was observed that there was no significant difference between the groups considering the time before (T0) and after treatment (T1), with p = 0.125 for H3 and p = 0.959 for H4 (Fig. 1).

### Methylation analyses

**Figure 2:**
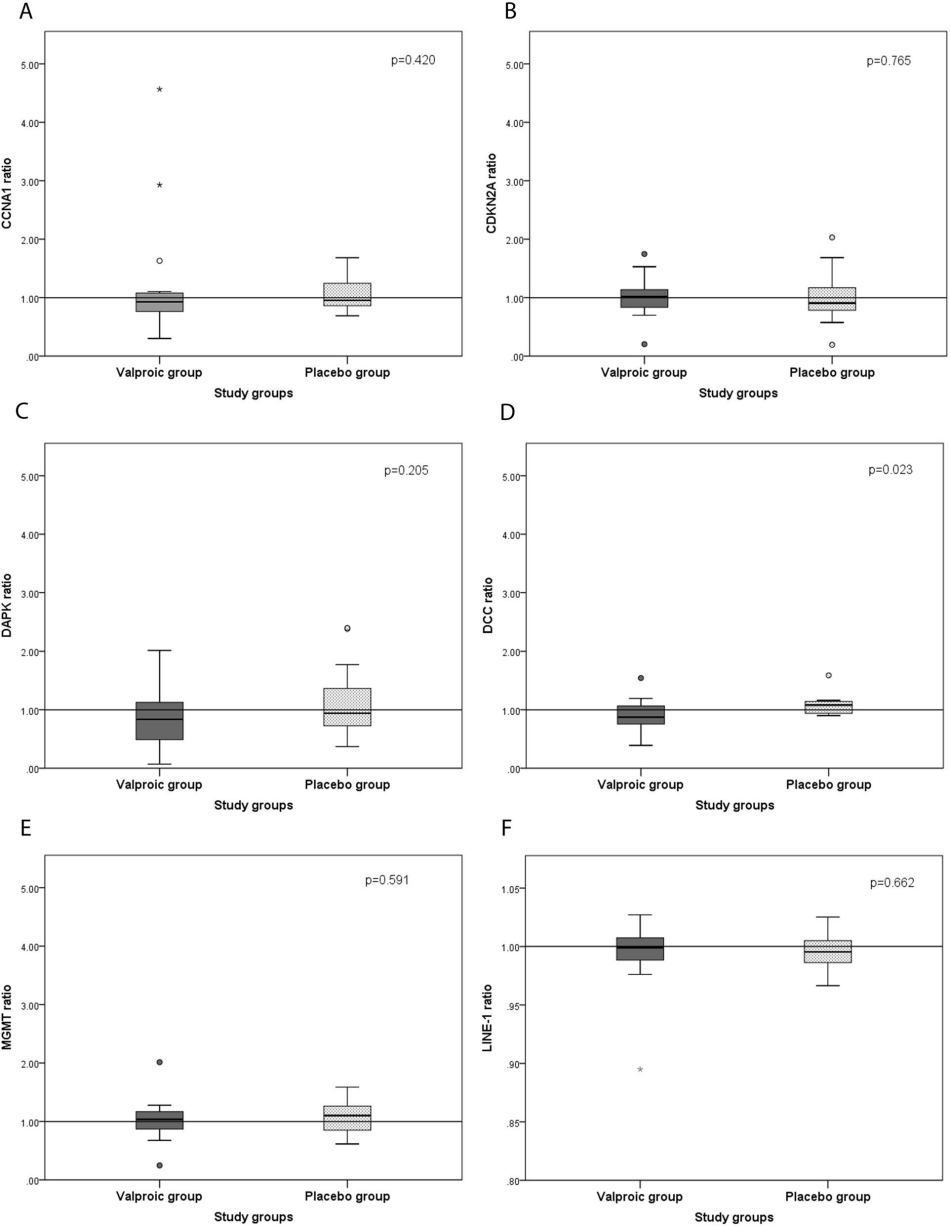
Comparison between the medians of the T1/T0 methylation ratios of *CCNA1* (**A**), *CDKN2A* (**B**), *DAPK* (**C**), *DCC* (**D**), *MGMT* (**E**), and LINE-1 (**F**) in oral rinse samples between the valproic and placebo groups. The boxplots show the median, maximum and minimum values, interquartile range and outliers (represented by circles and stars) in the two study groups. The dashed line passing through 1 indicates that the group participants located above the line demonstrated methylation.

The methylation profile of 5 genes was evaluated in the oral rinse DNA from 28 HNSCC patients (15 from the valproic group and 13 from the placebo group). In the valproic group, the methylation level of *DCC* was significantly lower in the posttreatment period than in the pretreatment period (p = 0.023). For *CCNA1* (p = 0.420), *CDKN2A* (p = 0.765), *DAPK* (p = 0.205) and *MGMT* (p = 0.591), and for the repetitive sequence LINE-1 (p = 0.662), which is normally hypermethylated to provide gene stability, there was no significant difference between groups considering the pretreatment (T0) and posttreatment times (T1). These results can be seen in Table 4 and Fig. 2.

**Table 4:** Medians of the methylation ratios of different genes in the posttreatment oral rinse (T1) in relation to the pretreatment time (T0) between the valproic and placebo groups.

Gene	Valproic group (n = 15)	Placebo group (n = 13)	p*
***CCNA1* methylation**			
Median (Min–Max)**	0.9 (0.3–4.5)	0.9 (0.6–1.6)	0.420
***CDKN2A* methylation**			
Median (Min–Max)	1.0 (0.2–1.7)	0.9 (0.1–2.0)	0.765
***DAPK* methylation**			
Median (Min–Max)	0.8 (0.0–2.0)	0.9 (0.3–2.4)	0.205
***DCC* methylation**			
Median (Min–Max)	0.8 (0.3–1.5)	1.0 (0.9–4.3)	**0.023**
MGMT*** methylation			
Median (Min–Max)	1.0 (0.2–2.0)	1.1 (0.6–1.5)	0.591
**LINE-1 methylation**			
Median (Min–Max)	1.0 (0.9–1.0)	1.0 (0.9–1.0)	0.662

*Calculated by the Mann–Whitney test. p-value < 0.05 is presented in bold.

**Min–Max = minimum–maximum.

***For *MGMT* dosage, 12 placebo samples were considered, since during one of the time points of one of the participants, it was not possible to evaluate methylation.

## Discussion

Methylation of the promoter region is a molecular mechanism for inactivating gene expression. It frequently occurs in the tumor suppressor genes. This epigenetic event plays a crucial role in the development of several tumors, including HNSCC (20). In HNSCC, aberrant methylation of CpG islands in the promoter region can affect genes involved in DNA repair, cell cycle regulation and apoptosis^16^, as well as those involved in cell differentiation^23^, proliferation^23^ and adhesion^24^. The detection of DNA methylation in body fluids such as oral rinses is a noninvasive technique that can be used to test cells in the epithelial layer of the mouth and pharynx^14^. Thus, the detection of aberrant DNA methylation or hypermethylation in body fluids such as oral rinse could have potential use for the development of biomarkers that may be beneficial in clinical practice^14^, as well as for evaluating the response to certain treatments^14^, as in the present study with valproic acid. In addition, the possibility of detecting molecular events even before the manifestation of clinical signs and symptoms^19^ could assist in managing the follow-up of these patients, making it possible to better select those who need closer active surveillance or for chemoprevention trials with the intention, in the long term, to improve survival and the incidence of second primary tumors in this population. In this study, the methylation status of *CCNA1, CDKN2A, DAPK, DCC* and *MGMT* was evaluated to verify whether their methylation profile was altered when patients were treated with valproic acid.

HNSCC patients in clinical follow-up presenting with hypermethylated genes in the oral rinse are more likely to develop recurrence^18,20^. Although patients without neoplasia were selected, aberrant methylation of the *CCNA1, DAPK, DCC* and *MGMT* genes was present in the pretreatment oral rinse samples in all participants of both groups, an epigenetic event that is the target of action of valproic acid. Thus, we expected to verify whether treatment with valproic acid would be able to alter the methylation signal of these mucosa samples represented by flaking cells present in the oral rinse of patients already out of treatment and without clinical or radiological evidence of neoplasia. Additionally, the acetylation or methylation signal in the pretreatment saliva was similar, regardless of the previous stage of the tumor, the allocated study group, alcohol consumption or the previous location of the HNSCC, since patients with a history of oral cavity cancer, regardless of whether the tumor was in the oropharynx, larynx or hypopharynx, were selected.

The presence of aberrant hypermethylation in the majority of patients in this trial raises doubts about the cause since, to date, after at least 4 years of clinical follow-up, the local, regional and distance recurrence rate is 26% for the entire population of the study, that of developing a second primary tumor is approximately 17%, and that of death 34%. In patients with no history of recurrence or a second primary tumor (the majority of the study population), the cancer field could be one of the causes of maintaining the hypermethylated state in the follow-up of patients with a history of HNSCC. Rettori et al.^20^ observed persistent hypermethylation of the *DCC* gene in the oral rinse collected at two posttreatment sessions and hypothesized that perhaps this hypermethylated state in the follow-up was related to physiological changes, such as chronic inflammation, widely dispersed in the mucosa of the upper aerodigestive tract in the population of patients with a history of HNSCC, in line with the theory of field cancerization^25^. Another reason could be the maintenance of smoking or alcohol consumption during the follow-up of these patients. It is worth mentioning that 35.3% of the patients in the valproic group and 29.4% of the patients in the placebo group were active smokers and that the methylation or acetylation signal may suffer from interference from agents such as tobacco. Care in selecting patients with a history of smoking is justified by the fact that the presence of epigenetic events such as aberrant methylation or deacetylation triggered by exposure to tobacco are essential for the action of valproic acid to manifest.

In this research, a significant reduction at DCC methylation pattern was observed in the valproic group. Some authors have shown that promoter region hypermethylation is the most common mechanism of inactivation of the *DCC* gene in patients with HNC and that this hypermethylation is present in up to 75% of studied HNSCC patients and in less than 1% of controls^17^. These reports aligned with the results of the current study suggest that *DCC* is a tumor suppressor gene that is epigenetically inactivated by hypermethylation of its promoter region in most patients with HNSCC and that its methylation signal could be reversed by the action of drugs with epigenetic action, such as valproic acid. The reason for the maintenance of the hypermethylated state of *DCC* in patients being followed for a previous history of HNSCC but without clinical signs of neoplasia needs further study for clarification, but it could correspond to the molecular status of the mucosa of patients with HNC, regardless of the presence of the tumor.

The authors of this study believe that the strength of the present research lies is the fact that biological validation took place in the context of a clinical, placebo-controlled trial, which also aimed to ascertain whether treatment with valproic acid is tolerable and feasible in this population, since despite being commercialized for many years, it was not designed for this purpose. In addition, based on these preliminary results, the authors believe that long-term treatment with this drug for chemopreventive purposes has great potential if safety, cost-effectiveness and therapeutic compliance are taken into account.

In terms of toxicity, despite the higher incidence of dyspepsia in the valproic group, there was no difference in adherence rates, which were greater than 90% in both groups. There was no difference in reporting this event 30, 60 or 90 days after the beginning of the use of the drug in the valproic group. However, as it was more frequently reported at the 30-day time point of the randomization than at the others, the dyspepsia event may have contributed to an increasing mean adherence in this group. Even so, it was observed that adverse events such as dyspepsia and somnolence are manageable, dose-dependent and of short duration and that they did not result in poor adherence or the need to interrupt the drug. In fact, the authors expected minor side effects related to valproic acid exposure based on the vast amount of medical experience in the use of this drug in the long term to treat neurologic and psychiatric conditions over the years of its existence^4^ and based on the experience of other authors^26^. This should definitively be highlighted, as an agent with chemopreventive intent has to be used for an undetermined period of time, and a drug with minor and manageable side effects could be better tolerated, leading to greater adherence.

The ability of valproic acid to inhibit HDAC in solid tumors has been verified with oral doses between 20 and 40 mg/kg^26^. In the present study, daily doses ranging from 1000 to 1500 mg/day were used, which is in line with those recommended by the literature to acetylate histones. It is important to emphasize that to achieve chemopreventive intent, a certain drug usually has to be administered at a high dose to reach a high serum concentration, leading to unacceptable side effects. That said, a physiologic dose of valproic acid administered with chemopreventive intent is promising, as it combines safety and tolerance in order to be used for years.

Some limitations of this study have to be addressed. The size of the study population may not have been sufficient to show differences in the patterns of gene methylation and acetylation of histones H3 and H4. In addition, other potential reasons for the inability to identify differences in methylation and acetylation between groups include the fact that the authors did not select only active smokers, who, in theory, would be subject to the epigenetic damage imposed by tobacco and that are the target of action by valproic acid; and the fact that the study was not restricted to a specific tumor subsite, prioritizing the oral cavity and oropharynx, where the presence of the methylation or acetylation signal could be better detected.

In the present study, the finding of a change in the methylation pattern of the *DCC* gene and the tendency of valproic acid to demethylate most of the selected genes and to acetylate H3 histones may enable the realization of new clinical trials for which the outcome is survival and the incidence of second primary tumors for chemopreventive purposes.
